# The Deadly Dog

**Published:** 1912-09

**Authors:** 


### The Deadly Dog

The town dog is an anachronism. He is out of date, out of place, a menace to health, life and limb, and a nuisance generally. In the tribal state of man he had his uses; perhaps was a necessity, as companion, watch dog and an aid to procuring sustenance. But in the present state of society he is worse than useless, and should be exterminated. Who has not been annoyed by the packs of yelping, snarling curs running at large in the streets when some miserable she dog is the center of attraction, a disgusting sight, especially for children to see; who has not been annoyed by the vicious, miserable little whelp that darts out from the yard or alley and runs after and snaps at every passing vehicle, wheel or auto?

These are annoyances. But the dog harbors the rabic poison, and is the principal source of hydrophobia. He makes Pasteur Institutes necessary. He harbors fleas,—perhaps infected. He is subject to skin disease,—perhaps infective. He harbors worms in his intestines and deposits them or their larvae on the garden truck and the lawns, whence they doubtless find their way into the intestines of man. And yet we, in our blind stupidity, permit him to breed, to propagate his kind in unlimited numbers, to be a source of annoyance and danger, and to compel the building of hospitals and Pasteur Institutes to be supported by a tax on the people. Further, it has been estimated that what he consumes, if properly economized and managed, would feed many families, and the cost of remedying the evils following in his trail would support the Christian ministry of the world.

It is claimed that the dog is necessary as a watch dog for the protection of the home. Did anyone ever hear of a yard dog biting a prowler or a burglar? or of biting anyone except a woman or a child? It is claimed for the dog that he is a pet for the children. Do the parents know the danger of such pet, if not the danger of hydrophobia,—always imminent,—then that of diphtheria, worms, mange, or worse? No; there is no place for the dog in the social scheme.

It may be said that this is too sweeping an indictment, and that I should exempt the "bird dog." It would be better for man if the birds were let alone; they are the farmers' friend, and no doubt save millions of dollars in crops. I should excuse, also, they say, the intelligent Colleys and New Foundlands. Why? We do not need them to herd sheep or rescue snow-bound pilgrims; they are just kept as pets, or ornaments. What right has a man to keep that on his premises which is a danger and a nuisance to others and to the public?

Now, no one loves a good dog better than I. He has many qualities that shame the average man, and I have often said, with a learned friend back in Virginia, where I came from, who paid me $50 for treating his imported pointer dog, "The more I see of men, the better I like dogs."

But I am not discussing this subject from a personal or sentimental viewpoint, but from a scientific one; it is a sanitary evil; the evil of an unlimited production of mongrels, a nuisance and a danger.

The hope of the utter extermination of the genus is Utopian.

It will come—with the further evolution of society. For the present we will have to compromise on the "valuable dogs"—and license them. They should be recognized and assessed and taxed as property, an *ad valorem* tax, and their owners should be required to place certain restrictions on them, and under no circumstances should they be permitted to run at large.

Here is another and a striking illustration of that perverseness—or stupidity—that seems to characterize all our dealings with disease, danger and injury. We seek to remedy the effects of evils that should not be permitted to exist. We treat results and ignore causes. As I have often remarked, we permit our children to burn their fingers, then put salve on it! We permit the defectives to breed in hordes and build palaces to house and care for them.

Exterminate the mongrel cur,—the principal source of hydrophobia. Tax him out of existence.

I extract the following from United States Public Health Reports No. 87 about Pasteur Institutes in the United States (made necessary largely by dog bites). Note that 44 of the 94 fatal cases reported are children under ten years of age:

"In 1908 there were twenty-three institutions in the country where this [Pasteur] treatment was administered; at present there are at least forty-two such institutions or their equivalent. In addition, there are five firms and public laboratories that furnish material for inoculations to practicing physicians, the business of these establishments having been established or their greatest activity having been developed within the last three years. The distribution of virus from the Hygienic Laboratory amounted to 443 treatments in 1908 and 942 in 1911. The number of persons known to have taken treatment in 1908 was about 1500, while the table shows 4625 for 1911. The decrease in the number of human deaths, coincident with an increase in the number of antirabic inoculations, at least effectually diposes of the claim still advanced occasionally by opponents of the Pasteur method that this treatment causes rather than prevents rabies.

"*Analysis of Fatal Cases in Man.*—Distribution as to age (data given in 94 cases): 1 to 10 years, 44 cases; 11 to 20 years, 15 cases; 21 to 30 years, 8 cases; 31 to 40 years, 9 cases; 41 to 50 years, 8 cases; 51 to 60 years, 2 cases; 61 to 70 years, 5 cases; 71 to 80 years, 2 cases; 81 to 90 years, 1 case. Total, 94 cases."

